# A solvent-free HPLC method for the simultaneous determination of Favipiravir and its hydrolytic degradation product

**DOI:** 10.1038/s41598-023-45618-x

**Published:** 2023-10-28

**Authors:** Yasmine Ahmed Sharaf, Mai H. Abd El-Fattah, Heba M. El-Sayed, Said A. Hassan

**Affiliations:** 1 Department of Analytical Chemistry, Faculty of Pharmacy, Zagazig University, Zagazig, 44519 Egypt; 2 Pharmaceutical Analytical Chemistry Department, College of Pharmaceutical Sciences and Drug Manufacturing, Misr University for Science and Technology, 6th of October City, Giza 12566 Egypt; 3 Department of Analytical Chemistry, Faculty of Pharmacy, Cairo University, Cairo, 11562 Egypt

**Keywords:** Diseases, Chemistry

## Abstract

During COVID-19 pandemic, Favipiravir (FPV) showed a great efficacy against COVID-19 virus, it produced noticeable improvements in recovery of the patients. The aim of this study was to develop a new, green and simple method for the simultaneous determination of FPV and its acid-induced degradation product (ADP) in its pure and pharmaceutical dosage forms. This method will be key for the inevitable development of FPV solution and inhaler formulations. A green micellar RP-HPLC method was developed using an RP-VDSPHERE PUR 100 column (5 µm, 250 × 4.6 mm) and an isocratic mixed micellar mobile phase composed of 0.02 M Brij-35, 0.1 M SDS and 0.01 M potassium dihydrogen orthophosphate anhydrous and adjusted to pH 3.0 with 1.0 mL min^−1^ flow rate. The detection was performed at 280 nm with a run time of less than six min. Under the optimized chromatographic conditions, linear relationship has been established between peak area and concentration of FPV and its ADP in the range of 5–100 and 10–100 µg mL^−1^ with elution time of 3.8 and 5.7 min, respectively. The developed method was validated according to the ICH guidelines and applied successfully for determination of FPV in its pharmaceutical dosage form.

## Introduction

In December 2019, COVID-19 was discovered in China and caused a global pandemic. About six different strains of Coronavirus were being reported to affect humans, even though, the seventh one which was identified as SARS-CoV-2 was similar to the SARS coronavirus genetically^1^. As soon as this pandemic has appeared, scientists and drug developers in all companies tried to develop new drugs and vaccinations to control this crisis and save peoples’ life. Novelty of virus’s strain made the old treatments and vaccinations ineffective against it, so there was an urgent need to develop new medications. Favipiravir (FPV) was one of the most effective drugs and made a great influence in the treatment.

Favipiravir (6-Fluoro-3-hydroxypyrazine-2-carboxamide) is a purine nucleic acid analog developed in Japan by Toyama Chemical for the treatment of viral infections. It is a pro-drug that is ribosylated and phosphorylated intracellularly to form its active metabolite Favipiravir ribofuranosyl-5'-triphosphate (FPV-RTP) that works by inhibiting RNA dependent RNA polymerase (RdRp) enzyme^2^. It is currently formulated as tablet dosage form.

Good results were achieved by the FPV treatment in patients with mild to moderate COVID-19 infections which was indicated by increased discharge rate and less mechanical ventilation^3,4^. It was also reported that patients who were treated with FPV showed a considerably higher viral clearance rate, after about 2 weeks from initiating FPV treatment^5^.

However, recent study showed a lack of positive effect of FPV on mortality, that could be attributed to the low plasma concentrations of FPV following oral administration^6^. Some adverse effects were noted with FPV such as diarrhea, changes in liver function parameters and hyperuricemia^7^. Accordingly, new dosage forms are being formulated for administration of FPV through lung inhalation^8^. Furthermore, there is an increasing need to formulate oral solution dosage forms suitable for children use^9^. Although being just preliminary studies without actual dosage forms in the market till now, these new formulations increased the importance of stability study of FPV especially in solutions. These studies will help optimize the formulation bioavailability and stability^10^. It’s well known that hydrolysis is the most common pathway for degradation in solutions; therefore, studying the hydrolytic degradation pathways of FPV is of utmost importance for these formulations to succeed and reach markets.

Green chemistry recently represents a universal trend, as a lot of studies are done in which many toxic chemicals and reagents are used, generating toxic waste products. Scientists and researchers should pay attention that these all-hazardous chemicals and wastes be replaced with more green, less toxic and eco-friendly ones. Paul Anastas and John Warner^11^ announced “The Twelve Principles of Green Chemistry” which are summarized PRODUCTIVELY. Simply, green chemistry is the usage of chemistry for contamination and toxic waste hindrance. Recently, green chemistry approach was extending to different analytical techniques such as electrochemistry, spectrophotometry, spectrofluorimetry and chromatography^12–17^.

HPLC technique is the optimum choice among analytical techniques for many pharmaceutical applications such as stability-indicating and assay of pharmaceutical dosage forms. The common presence of organic solvents is always a challenge to develop a green HPLC method. A simple and important mode of HPLC is Micellar liquid chromatography (MLC) which can be used for determination of drugs in pharmaceutical dosage forms and physiological fluids. In MLC separation of charged and uncharged solutes can be achieved using surfactants at a concentration exceeding their critical micelle concentration (CMC)^18^. MLC offers unique separation selectivity, high reproducibility, robustness, and safety; all these properties grabbed the attention of analysts for MLC^19^. Selection of surfactant in MLC is based mainly on their CMC. Surfactants with low CMC values are preferred as high CMC surfactants usually lead to a sticky mobile phase, which need higher pressure and may cause noise in the detector response^20^. Common surfactants used in MLC are sodium dodecyl sulphate (SDS), cetyltrimethylammonium bromide (CTAB), and polyoxyethylene 23 lauryl ether (Brij-35) which are known to be safe and biodegradable. In MLC, monomers of surfactants are adsorbed on the stationary phases thus altering their properties. In addition, they form micelles in the mobile phase that solubilizes the analytes. SDS is a commonly used anionic surfactant for retention of positively charged analytes, while Brij-35 is a non-ionic one. Usually, hybrid mobile phases are used in MLC where a small portion of organic modifiers (3‒15%) is used with the surfactants to solve the problem of slow mass transfer from the stationary phase^19^. This problem can also be solved by increasing temperature in the chromatography column to increases the mass transfer kinetics^21^, or using the non-ionic Brij-35 in combination with SDS^22^. The presence of Brij-35 can eliminate the need for presence of organic modifiers by decreasing stationary phase polarity. These facts make MLC a green procedure compared to traditional MLC and RP-HPLC.

According to the literature, some methods have been reported for determination of FPV including spectrophotometry^23,24^, spectrofluorimetry^25–27^, electrochemical^28–31^ and chromatographic methods^32–49^. Although stability studies have been reported for FPV^36,37,49^, they either lack the eco-friendly advantage, or use expensive instruments that are uncommon in QC laboratories (LC–MS).

The target of this manuscript is to progress a new simple, specific, and eco-friendly method for the simultaneous determination of FPV and its hydrolytic degradation product in its pure and pharmaceutical dosage form. The method can be further used in the development phase of FPV solution and inhaler formulations.

## Experimental

### Materials and reagents

All chemicals and reagents utilized throughout the experiments were all of analytical grade. FPV powder (100.03 ± 0.61%) was supplied from Marcyrl company. Brij35, SDS and Methanol HPLC grade were purchased from Sigma Aldrich (Germany), potassium dihydrogen orthophosphate anhydrous (98%) from Loba Chemie Pvt Ltd (India), sodium hydroxide was obtained from AVANTOR VWR Chemicals (USA) and hydrochloric acid from PIOCHEM (Egypt).

PIRAVAFI tablets, batch no. 2132642, labelled to contain 200 mg of FPV was purchased from local market (Marcyrl Pharmaceutical Industries, Egypt).

### Instruments

Chromatographic analysis was performed using Waters 2695 Alliance HPLC System with photodiode array (PDA), a quaternary, low-pressure mixing pump, autosampler and vacuum degassing. The detector is a PDA (model 2996) with a wavelength range of 190–800 nm and sensitivity settings from 0.0001 to 2.0000 absorbance units.

Mass spectrum was obtained using UPLC-MS/MS Waters 3100 (USA) with TQ detector, binary solvent manager pump, autosampler and MASS LYNX V4.1 software. IR Spectrophotometer: SHIMADZU 435 (Kyoto, Japan), sampling was undertaken as potassium bromide disks. pH meter (JENWAY) was used for pH adjustment.

### Chromatographic conditions

RP-C18 was used as stationary phase using VDSPHER PUR 100® column (5 µm, 250 × 4.6 mm). A solvent-free chromatographic separation was achieved using a mixed micellar mobile phase composed of 0.02 M Brij35 and 0.1 M SDS in presence of 0.01 M potassium dihydrogen orthophosphate anhydrous (pH 3 ± 0.01). pH was adjusted with orthophosphoric acid, and the elution was isocratic for 8.0 min. The flow rate was 1.0 mL min^–1^ while detection was done at 280.0 nm for FPV and its acid-induced degradation product (ADP). All Chromatographic separations were carried out at 40 ± 0.5 °C. Column was saturated with mobile phase at least 15 min before starting injection of samples, while at the end, column was washed with methanol then water for about 15 min each to confirm removing any residuals.

### Stock standard solution preparation

A stock standard solution of FPV (1 mg mL^−1^) was prepared by dissolving 25.0 mg of FPV in 10 mL deionized water, sonicated for 10 min then the volume was completed in 25.0 mL-volumetric flask with deionized water to obtain the desired concentration.

### Acid-induced degradation solution preparation

A certain weight of FPV (25 mg) was dissolved in 25.0 mL of 1.0 N HCl, refluxed for 2 h in water bath at 100 °C. The solution was neutralized after cooling with 1.0 N NaOH, the volume was completed with distilled water in a 100-mL volumetric flask to obtain ADP equivalent to 250.0 µg mL^−1^.

### Construction of calibration curve

From prepared stocks, different concentrations of FPV and ADP were prepared in the mobile phase and injected in triplicates. The procedure under chromatographic conditions was monitored. The peak areas were plotted against the corresponding concentrations and the regression equations were estimated. Regression equations for the developed methodology were established for FPV and ADP using 7 and 6 concentrations for FPV and ADP, respectively by plotting AUC obtained versus the corresponding concentration. For FPV, the concentrations used to build the calibration curve were 5, 10, 20, 40, 60, 80 and 100 µg mL^−1^, while for ADP 10, 20, 40, 60, 80 and 100 µg mL^−1^ were used.

### Application of the proposed HPLC method to PIRAVAFI tablets

Ten tablets were grinded into fine powder after removing their coats, an amount equivalent to 10.0 mg were accurately weighed, transferred into a 100-mL volumetric flask after that 50 mL methanol was added. The prepared solution was sonicated for 15 min, then it was completed to volume with distilled water. The solution was then filtered with 0.45 µm filter, and dilutions were done with the mobile phase to obtain concentrations within the linearity range. The procedure under chromatographic conditions was applied.

## Results and discussion

### Preparation of degradation product of FPV

According to previous studies, FPV is more liable to acidic degradation than other conditions^36,37,49^, so in this experiment acidic degradation was applied to obtain the hydrolytic degradation product of FPV. HCL (1.0 N) was added to FPV and refluxed in water bath at 100 °C, a sample was taken after different time intervals (1, 2 and 3 h), neutralized with NaOH, evaporated, and dissolved in methanol. To monitor the progress of degradation, solutions were spotted on TLC plates and developed using mobile phase of ethyl acetate: methanol: ammonia (2: 4: 0.1, by volume). Two spots were noticed after 1 h for FPV and the possible hydrolytic degradation product, but after 2 and 3 h one spot only for degradation was observed. For further confirmation, degradation solutions were injected into HPLC after 1 h showed a small peak at the same retention time of FPV but after 2 and 3 h, this peak completely disappeared. Therefore, the time for complete degradation of FPV was set at 2 h.

**Figure 1:**
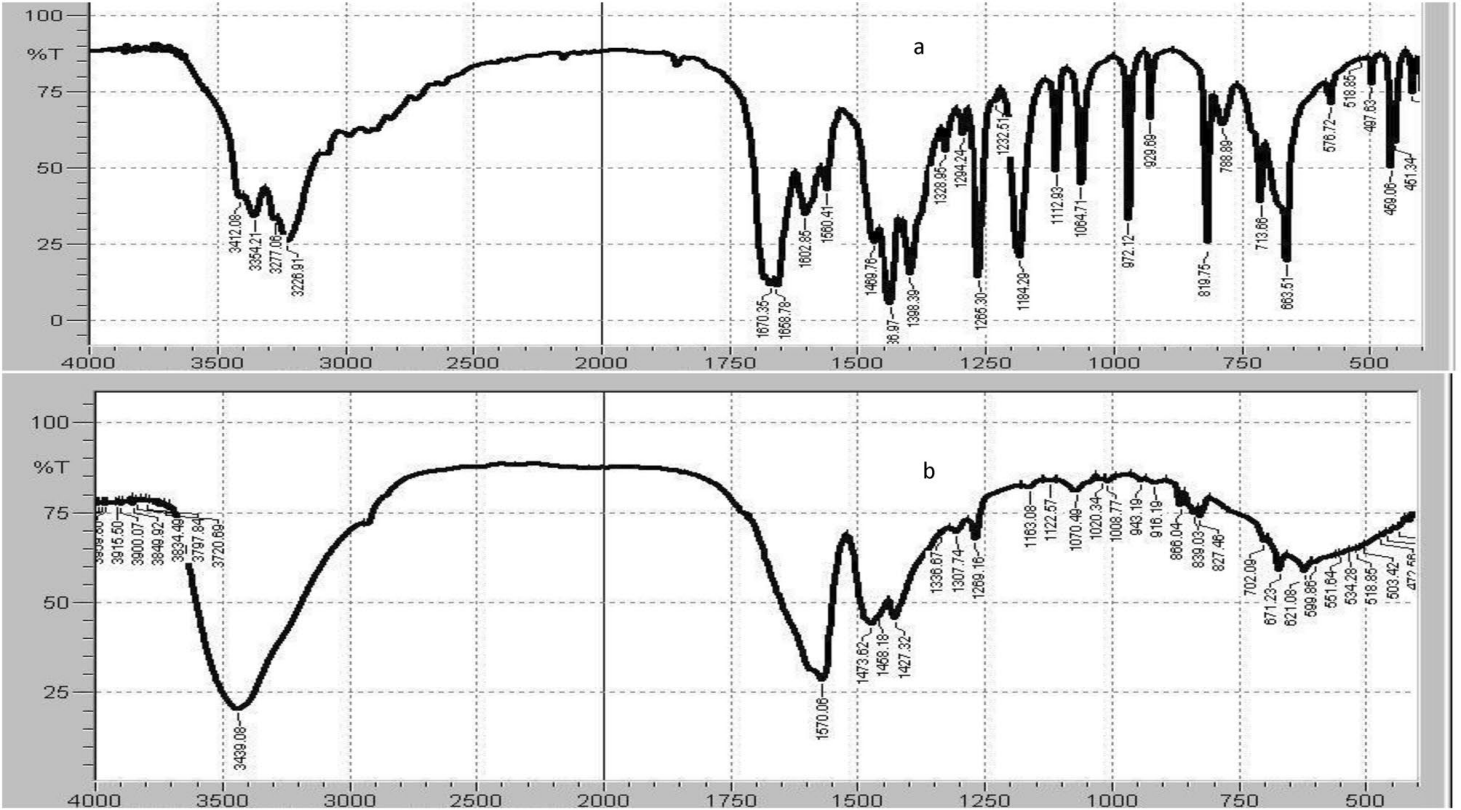
IR spectra of (**a**) Favipiravir (FPV) and (**b**) acidic degradation product (ADP).

FPV structure contains an amide group which may be subjected to hydrolysis in acidic media. To confirm this hypothesis FPV and ADP were subjected to IR and LC/MS. The IR spectrum of FPV showed forked peak at 3400 cm^−1^ which represents NH_2_ group of the amide (Fig. 1a). On the other hand, spectrum of ADP shows no forked peak of NH_2_ besides the appearance of broad peak of OH stretching at 3440 cm^−1^ characteristic to COOH group (Fig. 1b). Furthermore, the mass spectrum of ADP was obtained; it showed a peak at 157 m/z corresponding to ADP molecular weight The structure of the degradation product is not the main objective of our study. We focus on the stability indicating nature of the proposed method to help develop new solution dosage forms for FPV that will help treating COVID-19. However, our suggested structure is augmented with IR and mass spectra and the fact that it was already reported in literature. However, we will explain the findings that can be drawn from the mass spectrum in this reply but will not be added to the manuscript.

**Figure 2:**
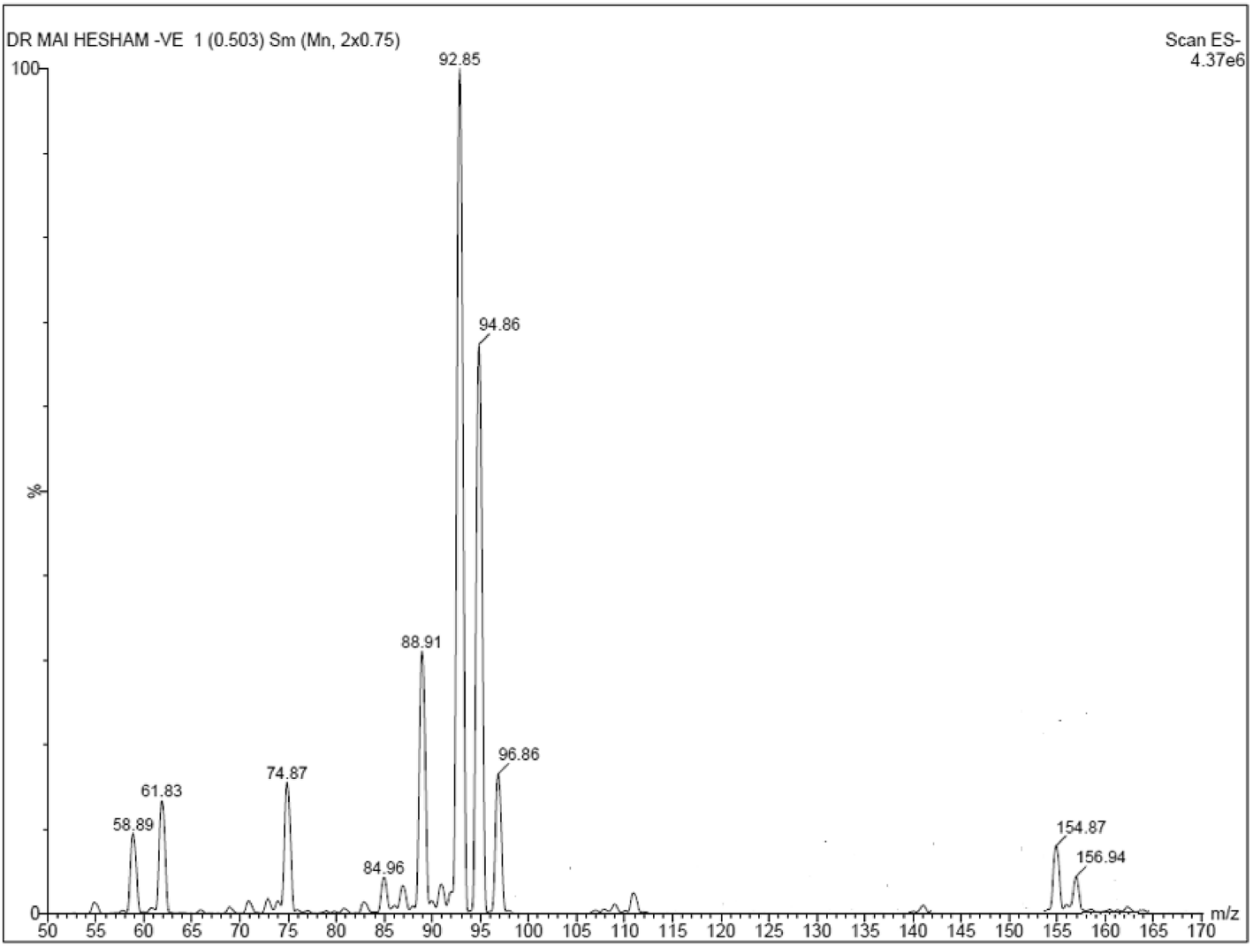
Mass spectrum of acidic degradation product (ADP) of Favipiravir.

The molecular ion peak corresponding to the degradation product itself showed a weak peak at m/z = 157 (M-1) despite having M.Wt. of 158, as ESI was operated in negative ion mode, Fig. 2.

**Figure 3:**
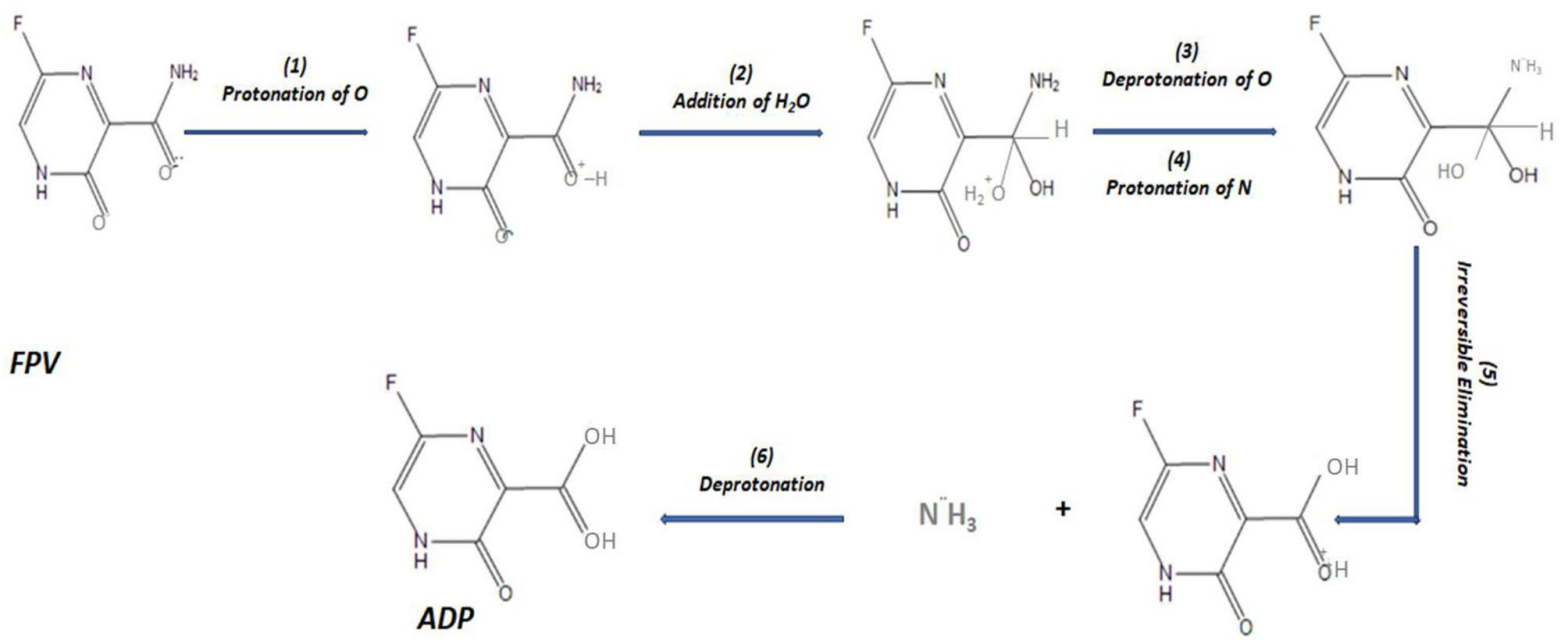
Suggested hydrolytic degradation pathway of Favipiravir.

This may suggest that FPV hydrolytic degradation is feasible through cleavage of the amide bond by protonation-addition-deprotonation-protonation-elimination-deprotonation (PADPED) mechanism, resulting in carboxylic acid and ammonium chloride salt^50^. Figure 3 shows the suggested degradation pathway of FPV.

### Optimization of chromatographic condition

To obtain high resolution in a short run time for the analyzed components with sharp symmetric peaks, there was an urgent need to study the effect of different factors affecting sensitivity, selectivity, and separation efficiency.

Different columns were tried to obtain the best resolution and separation. Symmetry C18 column (5 µm, 75 × 4.6 mm) and VDSPHERE PUR 100 C18-E (5 µm, 250 × 4.6 mm) column were used. The optimum peak resolution was obtained using the second column, so it was used for analysis of FPV and its ADP. On the other hand, different concentrations of mobile phase components were tried, Brij35 (0.01, 0.02, 0.03 M) and SDS (0.1, 0.12, 0.8 M) in 0.01 M potassium dihydrogen orthophosphate anhydrous were tried. A mobile phase composed of Brij35, SDS, and potassium dihydrogen orthophosphate anhydrous in the concentrations of 0.02: 0.1: 0.01 M gave the best results. As pH is an important parameter in HPLC and has a great effect on results including resolution and retention time, different pH were tested from (3–5) and the best results were obtained at pH 3. At pH below 3, peak of FPV was forked which may be due to protonation of amide group at high acidic medium.so no study was done below pH 3.

UV detection was done at different wavelengths 323, 227 and 280 nm, the best sensitivity was obtained at 280 nm at which measurements were done. Comparing between various flow rates 0.8, 1.0 and 1.2 the best results obtained at flow rate 1.0 ml min^–1.^

### Method validation

**Figure 4:**
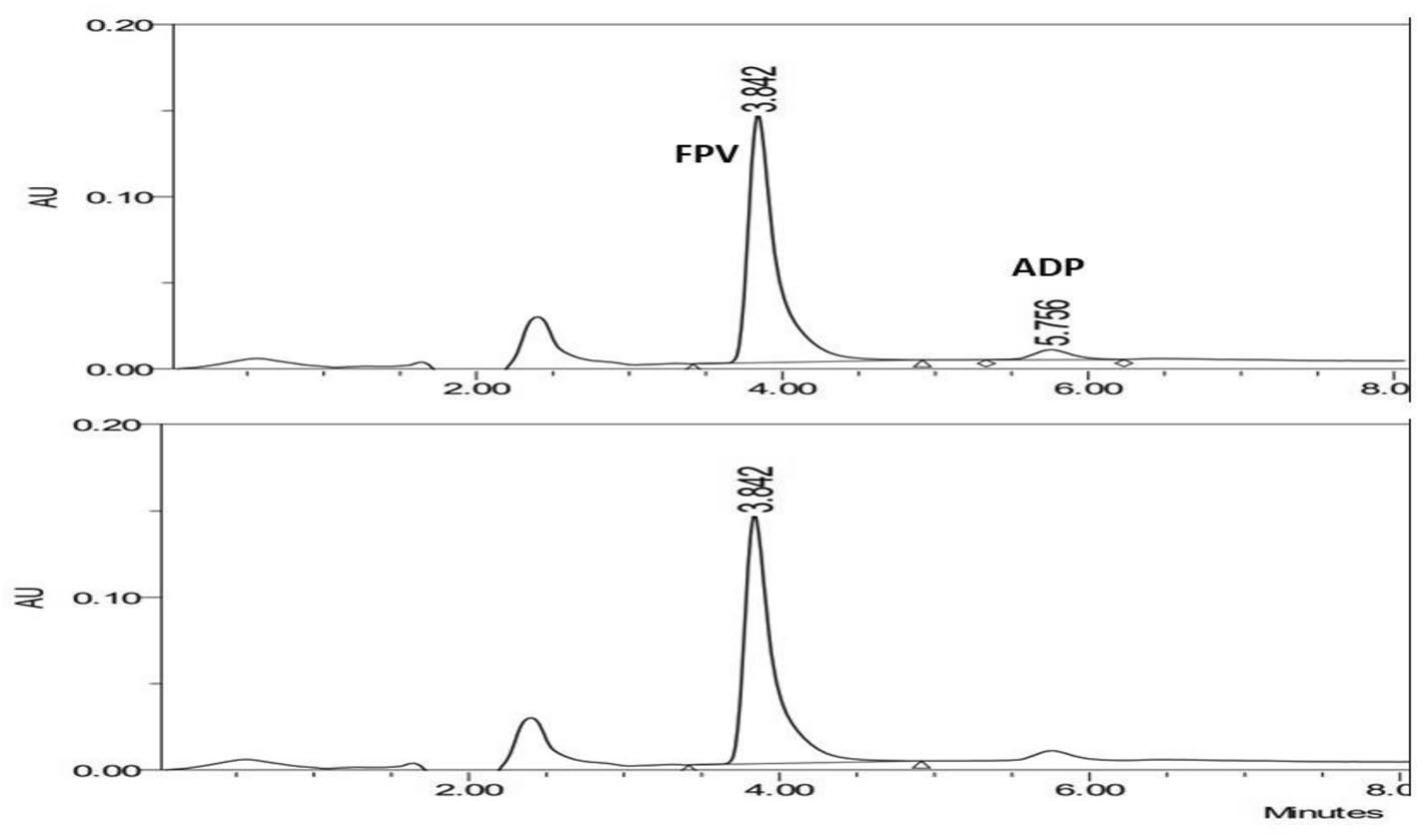
HPLC chromatograms of (**a**) synthetic mixture of Favipiravir (FPV) and its acid-induced degradation product (ADP) and (**b**) PIRAVAFI tablet dosage form (100 µg mL^−1^) at 280.0 nm.

The developed methodology were validated according to ICH guidelines^51^. Specificity of the developed method was confirmed by analysis of a mixture of FPV, and its ADP and comparing the results of pure FPV and its dosage form (Fig. 4). Regression equation for the developed methodology was established for FPV and ADP after plotting the area under curve (AUC) obtained versus the concentration of FPV and ADP. Linearity was obtained in the range of 5–100 and 10–100 µg mL^−1^ with correlation coefficients of 0.9999 and 0.9993 for FPV and ADP, respectively (Table 1). The correction factor was calculated for ADP and found to be 25.7^52^.

**Table 1:** Validation parameters data of the proposed HPLC method for Favipiravir (FPV) determination and its acid-induced degradation product (ADP).

Parameter	FPV	ADP
Linearity range (µg mL^−1^)	5–100	10–100
Slope	18,474	718.42
Intercept	17,771	6374.7
Regression coefficient (r)	0.9999	0.9993
Accuracy (mean ± SD)^a^	100.51 ± 1.20	99.61 ± 1.57
SD of residuals	7721.034	711.6004
LOD (µg mL^−1^)^b^	1.379	3.268
LOQ (µg mL^−1^)^b^	4.179	9.905
Intra-day precision^c^	0.428	1.075
Inter-day precision^d^	1.101	1.561

^a^Mean (*n* = 3) recovery % of five concentrations.

^b^Limits of detection “LOD” and quantitation “LOQ” are determined via calculations, LOD = 3.3 (SD of the residuals/slope), LOQ = 10 (SD of the residuals/slope).

^c^Intra-day precision (*n* = 3), three concentrations (40, 60 and 80 μg mL^−1^) repeated three times within the same day.

^d^Inter-day precision (*n* = 3), three concentrations (40, 60 and 80 μg mL^−1^) repeated three times in three successive days.

Accuracy of the developed method was tested and confirmed by calculating percentage recoveries (actual conc./claimed conc * 100) for five different concentrations in triplicate for each compound. Concentrations used for both FPV and ADP were 15, 30, 50, 70 and 90 µg ml^−1^. The closeness of the calculated percentage recovery results to the true values confirmed the accuracy of the method. Accuracy obtained was 100.51 and 99.61% for FPV and its ADP, respectively (Table 1). Repeatability (intra-day) and intermediate precision (inter-days) were established at three different measurements (40, 60 and 80 µg mL^−1^) within the same day and on three different days respectively, and acceptable values of RSD% were obtained that didn’t exceed 1.6% as presented in (Table 1).

Limits of detection [LOD] and quantification [LOQ] were calculated as 3.3 σ/S, and 10 σ/S, respectively, where (σ) is the residuals standard deviation and (S) is the slope of calibration curve, Table 1. There is urgent need to confirm and ensure that developed method can withstand small changes in the experimental conditions, thus robustness was assessed. Trials were performed to assess the impact of minor changes in concentrations of SDS and Brij-35 of the mobile phase. Changes in surfactant concentrations were (0.1 ± 0.02 M) and (0.02 ± 0.005 M) for SDS and Brij-35, respectively. By detecting changes in the AUC and retention times in terms of RSD%, it was within the acceptable limits as shown in Table 2. Short term stability was studied and results showed that FPV is stable for 24 h as shown in Table 2.

**Table 2:** Robustness and short-term stability of Favipiravir for the proposed HPLC method.

Parameters	RSD%	
	AUC	Retention time
SDS concentration (0.1 ± 0.02 M)	1.132	1.357
Brij-35 concentration (0.02 ± 0.005 M)	1.838	1.040
Short-term stability (24 h)	0.308	0.256

### System suitability parameters

Checking system suitability parameters of the proposed HPLC method was done to confirm that the system was working accurately during the experiment. Number of theoretical plates (N), heights equivalent to theoretical plates (HETP), tailing factor (T), capacity factor (K´), resolution and selectivity (α) were examined and were within the acceptable levels as shown in Table 3.

**Table 3:** System suitability parameters of the proposed HPLC methods for the determination of Favipiravir (FPV) and its acid degradation product (ADP).

Parameters	FPV	ADP	Reference values^53^
Retention time	3.842	5.756	
Number of theoretical plates(N)	2745	3583	> 2000
Tailing factor (T)	2.016	1.245	≤ 2
Capacity factor (K´)	0.921	1.878	**> **2.0
Height equivalent to theoretical plates (HETP)	0.0655 mm	0.0502 mm	
Resolution^a^	5.75		> 2
selectivity factor (α)^a^	2.086		> 1

^a^Resolution and selectivity factor are determined between the peaks of FPV and ADP.

### Application to pharmaceutical dosage form

The developed analytical method was used for determination of FPV in PIRAVAFI tablets. Calculated percentage recovery of FPV was statistically comparison to the reported method^49^. Student’s t-test and F-test showed no significant difference among the results (Table 4), proving the proposed method can be successfully applied for analysis of FPV dosage forms in QC laboratories.

**Table 4:** Statistical analysis of the proposed HPLC method and the reported method for the analysis of PIRAVAFI tablets.

Parameters	Reported method^54^^a^	Proposed method
Mean recovery %^b^	99.405	98.890
Variance	0.338	0.429
n	4	4
Students t-test^c^	–	1.173 (2.44)
F-test^c^	–	1.26 (9.276)

^a^Inertsil ODS-3V C18 maintained at 30 °C, using Phosphate buffer (pH 3.5) and acetonitrile, (90:10, v/v) as mobile phase with flow rate of 1.0 mL min^−1^ at 358.0 nm.

^b^Average of three determinations.

^c^Values between parenthesis are corresponding to the theoretical values of *t* and *F* (*P* = 0.05).

## Comparison with other reported methods

Regarding greenness assessment, developing environmentally friendly analytical methods has become a universal issue, greenness of the developed method and five reported methods was evaluated using two greenness assessment techniques: the green analytical procedure index (GAPI)^55^ and the AGREE metric^56^. GAPI assessments includes three parts firstly, sample preparation which involves the steps, transportation, and organic solvents. Secondly, the quantity of reagents and their National Fire Protection Association score. The third part takes instrument and waste into consideration.

As shown in Table 5, GAPI pictogram of the proposed method has only two red zones that stand for the offline sampling and no waste treatment which represents no problem as all reagents used were eco-friendly and totally green. Assessment of the proposed method was the same as the two reported green methods^42,47^ which offered no stability study. On the other hand, the reported methods^48,49,54^ are less eco-friendly as shown in the upper left part with red colors and upper right part with yellow colors related to using organic solvents, so their safety and toxicity is more compared with the green color in both parts of the proposed method pictogram.

**Table 5:** Comparison between the proposed micellar HPLC and the reported methods for analysis of Favipiravir (FPV) and its acid degradation product (ADP).

	Proposed method	Reported method^42^	Reported method^47^	Reported method^48^	Reported method^49^	Reported method^54^
Technique	Micellar HPLC–UV	Micellar HPLC–UV	Micellar HPLC–UV	HPLC–UV	HPLC–DAD	RP-HPLC
Linearity range (µg ml^−1^)	FPV: (5–100) ADP: (10–100)	FPV: (0.5–50.0)	FPV: (10–100)	FPV:(10–100)	FPV:(6.25–250.00)	FPV:(50–250)
Organic solvent	Totally free	Totally free	Totally free	Phosphate buffer (pH 3.2) and acetonitrile, (90:10, v/v)	Phosphate buffer (pH 3.5) containing 0.1% heptane, sulphonic acid sodium salt, methanol and acetonitrile, (62:28:10, v/v)	Phosphate buffer (pH 3.5) and acetonitrile , (90:10, v/v)
Run time (min)	8.0	5.0	4.0	8.0	10.0	15.0
Column	RP-VDSpher PUR 100	RP-C18 core–shell	VDSpher-150 C18-E	Inertsil ODS-3V C18	Zorbax C18	Inertsil ODS-3V C18
LOD (µg ml^−1^)	FPV: 1.379 ADP: 3.268	0.040	0.985	1.200	1.020	2.186
LOQ (µg ml^−1^)	FPV: 4.179 ADP: 9.905	0.120	2.986	3.600	3.100	6.626
Application	Stability assessment of FPV and ADP in pharmaceutical dosage form	Molnupiravir and FPV in their dosage forms	Bulk powder	Bulk powder	Stability assessment of FPV in its tablet dosage form	Stability assessment of FPV in its tablet dosage form
GAPI						
AGREE						

AGREE is represented as a clock-shaped chart with a perimeter divided into 12 sections, each corresponding to one of the Green Analytical Chemistry principles with color scale (red-yellow-green). The AGREE pictogram has an overall evaluation color indicating the final score, performance of the analytical procedure in each criterion as well as an overall assessment score on a scale from 0 to 1. As shown in Table 5, the high score of the proposed method (0.84) confirms high greenness of method. The two reported methods^42,47^ have the same greenness score of the proposed procedure, but lacking quantification and separation of ADP mentioned in this study. Comparing with the three other reported methods^48,49,54^, they have low AGREE score as presented by the red color at Sect. (12) corresponding to hazardness, corrosiveness, toxicity and flammability of reagents used. In addition, yellow color at Sect. (10) which represents the type of reagents used compared with the a green section of the proposed method.

Considering other method characteristics, all the previously reported methods used either organic solvents as mobile phase^48,49,54^ or green solvents^42,47^, but none of them included offered quantitation of the degradation product.

## Conclusions

Simple, accurate and eco-friendly HPLC method was developed and validated for the simultaneous determination of FPV and its hydrolytic degradation product. The developed method was successfully applied to marketed pharmaceutical dosage forms. The method can be used for the development of solutions or inhalers dosage forms for FPV. These dosage forms are urgently needed to increase its efficacy in treatment of SARS-CoV-2 viral infection. The greenness of the proposed method was estimated using GAPI and AGREE metrics and proved its low ecological impact and made it preferred to other reported methods to be applied in quality control laboratories.

## Data Availability

The data analyzed during the current study are available from the corresponding author on reasonable request.
